# A Novel Hybrid Model for Drawing Trace Reconstruction from Multichannel Surface Electromyographic Activity

**DOI:** 10.3389/fnins.2017.00061

**Published:** 2017-02-14

**Authors:** Yumiao Chen, Zhongliang Yang

**Affiliations:** ^1^Fashion and Art Design Institute, Donghua UniversityShanghai, China; ^2^College of Mechanical Engineering, Donghua UniversityShanghai, China

**Keywords:** drawing trace, surface electromyography, gene expression programming, regression, muscle computer interface

## Abstract

Recently, several researchers have considered the problem of reconstruction of handwriting and other meaningful arm and hand movements from surface electromyography (sEMG). Although much progress has been made, several practical limitations may still affect the clinical applicability of sEMG-based techniques. In this paper, a novel three-step hybrid model of coordinate state transition, sEMG feature extraction and gene expression programming (GEP) prediction is proposed for reconstructing drawing traces of 12 basic one-stroke shapes from multichannel surface electromyography. Using a specially designed coordinate data acquisition system, we recorded the coordinate data of drawing traces collected in accordance with the time series while 7-channel EMG signals were recorded. As a widely-used time domain feature, Root Mean Square (RMS) was extracted with the analysis window. The preliminary reconstruction models can be established by GEP. Then, the original drawing traces can be approximated by a constructed prediction model. Applying the three-step hybrid model, we were able to convert seven channels of EMG activity recorded from the arm muscles into smooth reconstructions of drawing traces. The hybrid model can yield a mean accuracy of 74% in within-group design (one set of prediction models for all shapes) and 86% in between-group design (one separate set of prediction models for each shape), averaged for the reconstructed x and y coordinates. It can be concluded that it is feasible for the proposed three-step hybrid model to improve the reconstruction ability of drawing traces from sEMG.

## 1. Introduction

Drawing is one of the oldest forms of human expression and communication, predating written language by thousands of years (Tversky, 2011). It can be used to express one's creativity, and therefore has been used extensively in the fields of art, design, science and so on. As computers grow more powerful and widely available, drawing instruments have evolved from traditional pen and paper to input devices such as mouse, digital pen, touch medium tablet, interactive pen display and touch screen. However, some researchers have found that computer-based drawing system can inhibit the variety of design ideas, unlike sketching on paper which resulted in increased variety (Goel, 1995). It is causing some user-interface (UI) researchers to look at ways to develop more accessible, natural and people-oriented human-computer interfaces (HCI) (Landay and Myers, 2001; Kara and Stahovich, 2005). As is known to all, drawing is a complex interplay between the nervous system and the neuromuscular activities of the upper extremity (Okorokova et al., 2015). Thus, the technology of muscle-computer interfaces (MCI) can be applied in the design of gesture based more accurate and interactive digital drawing tool (Chowdhury et al., 2013; Silva et al., 2015). However, the study on the MCI of drawing is still in its infancy.

As a new interaction style mediated by physiological data (Fairclough, 2009; Silva et al., 2015), MCI mainly involves surface electromyography (sEMG) signals (Saponas et al., 2008; Chowdhury et al., 2013). The sEMG is a non-invasive method to register the electrical activity of the muscle fibers during a motor task, as triggered by the impulses of activation of the innervating motor neurons (Farina et al., 2014; Okorokova et al., 2015). Recently, a variety of sEMG based interfaces have been developed for rehabilitation (Wang et al., 2015), hand motion recognition (Ding et al., 2015) and reconstruction (Fernandez-Vargas et al., 2016), prosthesis control (He et al., 2015; Zhang et al., 2016), sign languages recognition (Cheng et al., 2015), facial expression recognition (Chen et al., 2015; Geangu et al., 2016), movement recognition of upper and lower limbs (Tang et al., 2014; Young et al., 2014), muscle fatigue analysis (Hawkes et al., 2015).

After 60 years of development (Battye et al., 1955; Englehart et al., 2000; Li et al., 2010; Farina et al., 2014; Dosen et al., 2015), the focus of the research in using sEMG signals as a control source for intelligent exoskeletons and prostheses has come to tackle practical problems, such as electrode shift (Hargrove et al., 2008; Stango et al., 2015), real-time operation (Kuiken et al., 2009; Fougner et al., 2014), signal non-stationarity (Lorrain et al., 2011), load variation (Tang et al., 2015), low cost (Brunelli et al., 2015), and force variation (Scheme and Englehart, 2011), for improving the clinical applicability of sEMG-based exoskeletons and prostheses. In contrast, sEMG-based gesture recognition techniques used for identifying subtle gesture traces (Kristensson and Zhai, 2004), such as handwriting and drawing, are still relatively far away from practical applications.

The existing research has demonstrated the feasibility of decoding handwriting solely from sEMG signals (Linderman et al., 2009). Two fundamental approaches have been proposed for decoding handwriting from the EMGs. In the first approach, several papers addressed the question of written character recognition based on sEMG, which involved the implementation of pattern recognition techniques to distinguish between muscle activation patterns for different written characters (Okorokova et al., 2015). The recognition performance attained with sEMG based methods is comparable to that achieved by computer-vision based methods of written character recognition (Linderman et al., 2009; Asano and Honda, 2010; Huang et al., 2010; Chihi et al., 2011; Li et al., 2013; Shih et al., 2016). In the second approach, several researchers considered the problem of reconstruction of handwritten traces from multichannel EMG activity, which involved implementation of regression techniques to reconstruct X-coordinate and Y-coordinate of handwritten traces from EMGs using a linear or non-linear model (Linderman et al., 2009; Okorokova et al., 2015). However, to date, decoding sEMG signals with algorithms to extract and reproduce drawing traces has not so far been explored.

Reconstructing drawing traces from sEMG recordings is important for both theoretical and practical reasons, because drawing not only precede written language but also served as the basis for it in human culture (Gelb, 1963; Tversky, 2011). Once we learn how to model the relationship between EMG signals and drawing traces, we can introduce this knowledge to many rapidly expanding fields and practices, including computer-aided design, 3D printing, virtual reality, neural engineering, rehabilitation engineering, biomedical engineering, robot control, as well as human-machine interfaces in general.

As the study on drawing trace reconstruction from sEMG signals is still in its infancy, advance algorithms are urgently needed for this to be possible (Englehart and Hudgins, 2003; Nielsen et al., 2011). Linear regression methods, including the Wiener filter (Linderman et al., 2009) and the Kalman Filter (Okorokova et al., 2015), have been utilized for the reconstruction of handwriting from multichannel EMG activity with some success. However, the nonlinearity of the relation between the sEMG signals and the subtle gesture traces prompts to explore the use of non-linear models in this application (Okorokova et al., 2015). This, however, seems to be an ideal situation for the application of Gene Expression Programming (GEP), which are able to develop a non-linear EMG-trace prediction model, produce simple explicit formulations with high accuracy and reduce the number of EMG features (Ferreira, 2006; Zhang and Sun, 2013).

In our previous study (Yang and Chen, 2016), we proposed an sEMG-based method using two analysis windows and GEP for the recognition of 11 basic one-stroke shapes from sketching in conceptual design. The average recognition rate for the 11 basic one-stroke shapes achieved by the GEP classifier was more than 96%. Therefore, discrete symbol recognition from sEMG signals has been a relatively easy task. In contrast, a much more challenging task is to reconstruct drawing traces from sEMG signals, which is critical to a novel and natural interactive paradigm that enables people to be more creative, expressive and satisfied in their daily lives.

This study proposes a three-step hybrid model for the reconstruction of drawing traces based on multichannel sEMG signals. In order to verify the validity and robustness of our method, we selected 12 basic one-stroke shapes during drawing. An experiment protocol was established to record the sEMG signals from 4 forearm and 3 upper arm muscles of 5 participants who were instructed to draw on a digitizing table with a pen while tracing and covering each printed one-stroke shape on a template. The main idea behind our method is to combine three-step algorithms for drawing trace reconstruction. The first step is coordinate state transition, the second step is feature extraction of sEMG signals, and the third step is to construct a non-linear EMG-trace prediction algorithm derived by GEP. In addition, we compared the performance of the three-step hybrid model to that of a Kaman Filter.

## 2. Methods

### 2.1. The three-step hybrid model

#### 2.1.1. The schematic procedure of the hybrid model

**Figure 1:**
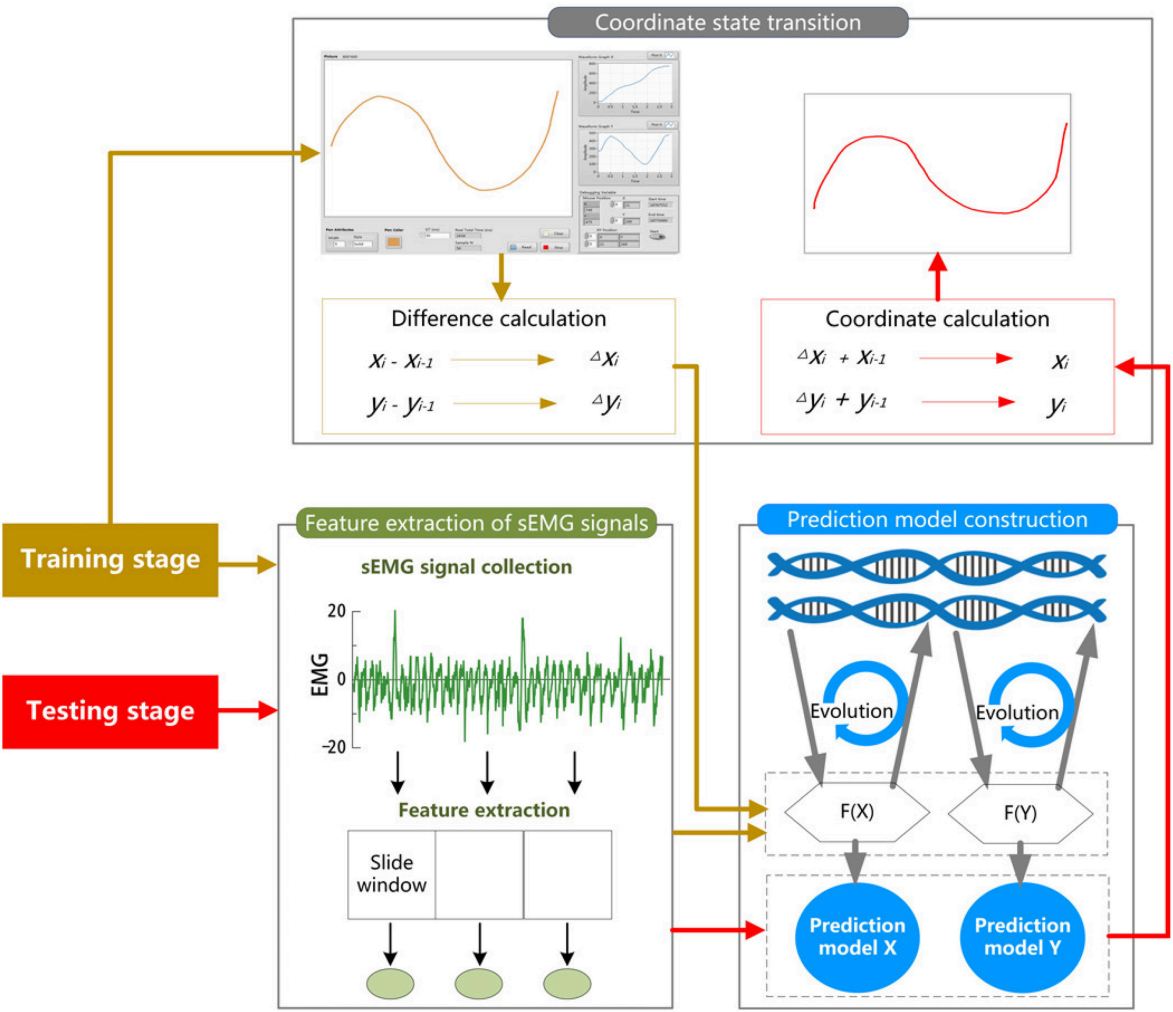
**The schematic procedure of the three-step hybrid model**. For each participant of the experiment, the trials are randomly divided into two subsets, a training set and test set. During training stage (golden), the training trials are used for learning and constructing two prediction models from the data of the coordinate state transition and feature extraction of sEMG signals based on GEP. During the testing stage (red), the extracted features of sEMG data from the testing trials and the constructed prediction models are then used for the prediction of the X-coordinate and Y-coordinate.

In this paper, we develop a three-step hybrid drawing trace reconstruction approach based on sEMG signals. This method has three steps for the training and testing stage respectively. The schematic procedure of the three-step hybrid model is illustrated in Figure 1.

During the training stage, in the first step, the x and y coordinates are collected and converted to the differences between the present coordinate state and the previous state. In the second step, sEMG signals are simultaneously recorded, and the features of them are extracted. In the third step, the differences of x and y coordinates are set as the target vector and the features as the input vectors for constructing two non-linear EMG-trace prediction models with GEP.

During the testing stage, in the first step, the features of sEMG signals for testing are extracted. In the second step, the features are used to calculate the differences of x and y coordinates via the corresponding prediction models derived by GEP. In the third step, the estimates of the x and y coordinates are computed recursively from the output vectors of the GEP-based prediction model.

#### 2.1.2. Coordinate state transition

We will collect the x and y coordinates during each drawing trial every 50 ms. By a trial, we define a recording epoch during which a subject draws a single shape. During the training stage, we will calculate the differences between the *i*th coordinate state and the *i* − 1th coordinate state as the target vector of GEP. The differences of x and y coordinates can be computed as:

(1) $$\begin{array}{l} \Delta x_{i}=x_{i}-x_{i\;-\;1}\;i=1,2,3,\ldots ,n \end{array}$$

(2) $$\begin{array}{l} \Delta y_{i}=y_{i}-y_{i\;-\;1}\;i=1,2,3,\ldots ,n \end{array}$$

where *x*_*i*_ represents the *i*th x-coordinate, *y*_*i*_ represents the ith y-coordinate, and *n* represents the total amount of coordinates.

During the testing stage, we will calculate the predicted x and y coordinates from the outputs of GEP model recursively as follows:

(3) $$\begin{array}{l} {\hat{x}}_{i}=\Delta {\hat{x}}_{i}+{\hat{x}}_{i\;-\;1}\;i=1,2,3,\ldots ,n \end{array}$$

(4) $$\begin{array}{l} {\hat{y}}_{i}=\Delta {\hat{y}}_{i}+{\hat{y}}_{i\;-\;1}\;i=1,2,3,\ldots ,n \end{array}$$

where the starting pen location point (${\hat{x}}_{0}$,$\hat{y}$_0_) is set to the x and y coordinates of the start point of each stroke, $\Delta {\hat{x}}_{i}$ represents the *i*th predicted difference of x-coordinate, and Δ$\hat{y}$_*i*_ represents the *i*th predicted difference of y-coordinate.

#### 2.1.3. Feature extraction of sEMG signals

To be consistent with the collected coordinates and calculated coordinate differences in the time dimension, all sEMG data will be segmented for feature extraction using the adjacent windowing techniques (Englehart and Hudgins, 2003; Oskoei and Hu, 2007). The analysis windows have a duration of 50 ms for feature extraction, and the successive analysis windows are adjacent and disjoint. The onset of one drawing trial is designated as the onset of feature extraction. As a time domain feature, the Root Mean Square (RMS) represents the characteristic of the amplitude change of EMG signals on the time dimension, which can nondestructively measure the state of muscle activity in real-time (Yang et al., 2016). The RMS is also widely accepted (Shrirao et al., 2009; Tang et al., 2015). It is therefore adopted in this study as below (Chen et al., 2016):

(5) $$\begin{array}{l} RMS=\sqrt{\frac{1}{N}\overset{N}{\underset{i=1}{\sum }}V_{i}^{2}} \end{array}$$

where *v*_*i*_ is the voltage at the *i*th sampling and *N* is the number of sampling points.

#### 2.1.4. Prediction model construction based on the GEP

GEP is a new technique of evolutionary algorithm for data analysis (Zhou et al., 2003). It was first invented by Ferreira (2002), and is a development of Genetic Programming (GP). GP was first proposed by Koza (1992), as a generalization of Genetic Algorithms (GAs) (Mitchell, 1998). GEP is, like GAs and GP, a genetic algorithm as it uses populations of individuals, selects them according to fitness, and introduces genetic variation using one or more genetic operators (Ferreira, 2002). The fundamental difference between the three algorithms resides in the nature of the individuals (Ferreira, 2006). GEP uses fixed-length, linear strings of chromosomes (genotype) to represent programs in the form of expression trees (phenotype) of different shapes and sizes, and implements a GA to find the best program (Zhou et al., 2003). GEP uses the same kind of diagram representation of GP, but the entities evolved by expression tree are the expression of a genome. In GEP, the individuals are non-linear structures of different size and shape (expression trees) that are encoded by linear chromosomes composed of multiple genes, each gene encoding a smaller subprogram (Landeras et al., 2012). The individuals are often selected and copied into the next generation based on their fitness by roulette-wheel sampling with elitism (Ferreira, 2006). This guarantees the survival and cloning of the best individual to the next generation.

Compared with other non-linear models, the GEP model has many advantages. First of all, on account of the characteristics of simplicity, high efficiency, and functional complexity, GEP combines the advantages of both GAs and GP, while overcoming some of their limitations, which offers great potentiality to solve complex modeling and optimization problems (Zhou et al., 2003). Moreover, after the training process, GEP can produce simple explicit formulas with high accuracy (Landeras et al., 2012) and reduce the number of sEMG features (Yang et al., 2016). In addition, our proposed GEP model showed promise for recognizing sketching based on sEMG signals (Yang and Chen, 2016). Thus, GEP can be used here for symbolic regression or function finding. The detailed theoretical information about GEPs can be found in the papers mentioned above.

In our work, the procedure of derivation of two prediction models of x and y coordinates based on the application of GEP is as follows:

##### 2.1.4.1. Step 1: the selection of fitness function

For this problem, the maximum fitness (*f*_*max*_) was set to 1,000, and then the fitness function (*f*_*i*_) of an individual program (i) is expressed as follows:

(6) $$\begin{array}{l} f_{i}=1000*\frac{1}{RMSE_{i}+1} \end{array}$$

(7) $$\begin{array}{l} RMSE_{i}=\sqrt{\frac{1}{m}\overset{m}{\underset{j=1}{\sum }}\left(F_{ij}-T_{j}\right)} \end{array}$$

where *RMSE* is the root mean square error, *m* is the total number of fitness cases, *F*_*ij*_ is the value predicted by individual program *i* for fitness case *j*, and *T*_*j*_ is the target value for fitness case *j*. For a perfect fit, *F*_*ij*_ = *T*_*j*_.

##### 2.1.4.2. Step 2: the creation of the chromosomes

This step consists of choosing the set of terminals *T* and the set of functions *F* to create the chromosomes. In the current problem, the terminal set includes the following variables: *T* = *d*_1_, *d*_2_, *d*_3_, *d*_4_, *d*_5_, *d*_6_, *d*_7_, representing 7 input dimensions of RMS values extracted from 7 channels of sEMG signals. The choice of the appropriate function depends on the viewpoint of user. In this study, six elements were chosen as the mathematical function set: *F* = +, −, ×, ÷, *Sqrt, Exp, Inv, x*^2^, *Sin, Cos*.

##### 2.1.4.3. Step 3: the selection of the chromosomal architecture

In this study, length of head, *h* = 15, length of tail, *t* = 16, and six genes per chromosomes will be employed.

##### 2.1.4.4. Step 4: the selection of the linking function

This step is to choose the linking function, which will be addition for this study. Here, the sub expression trees (ET) are linked by addition.

##### 2.1.4.5. Step 5: the selection of the GEP operators

The learning algorithms of GEP apply the following basic operators: mutation, inversion, one-point recombination, two-point recombination, gene recombination, gene transposition, Insertion Sequence (IS) transposition and Root Insertion Sequence (RIS) transposition (Landeras et al., 2012).

**Figure 2:**
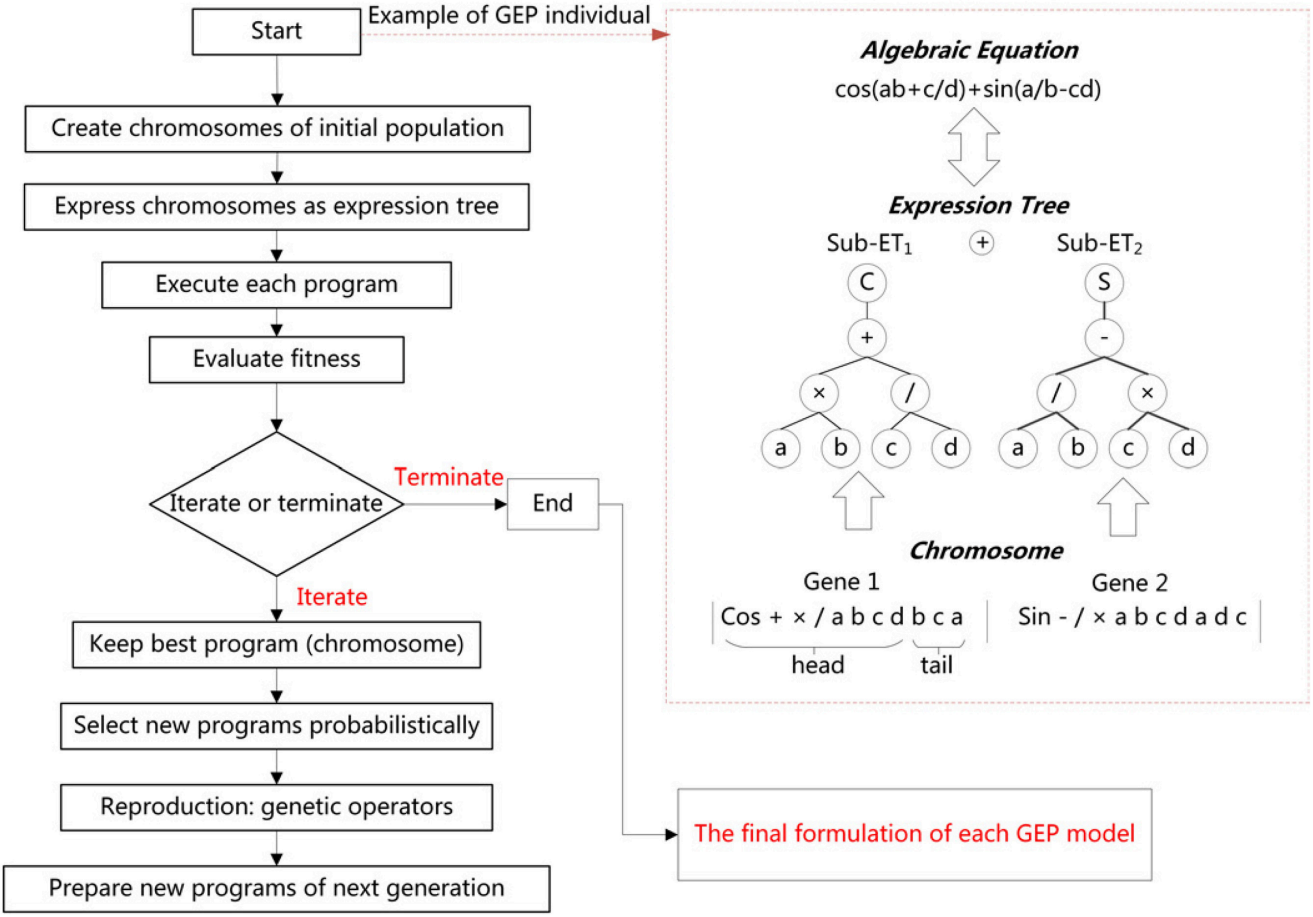
**The general GEP structure**.

In Figure 2 there is a description of the GEP implementation procedure described above. Table 1 shows various parameters involved in the GEP algorithm per run. GeneXproTools 5.0 software package was used for the implementation of GEP models.

**Table 1:** **Training parameters settings for the GEP algorithm**.

**Parameter**	**Value**
Number of chromosomes	200
Function set	*F* = +, −, ×, ÷, *Sqrt, Exp, Inv, x*^2^, *Sin, Cos*
Terminal	*T* = *d*_1_, *d*_2_, *d*_3_, *d*_4_, *d*_5_, *d*_6_, *d*_7_
Number of genes, head size, gene size	6,15,31
Linking function	Addition
Fitness function error type	Root Mean Square Error (RMSE)
Mutation rate	0.0014
Inversion rate	0.0055
IS/RIS/gene transposition rate	0.0055
One-point/two-point recombination rate	0.0028
Gene recombination rate	0.0028
Termination criterion	Generation (2,000) or max fitness

### 2.2. Performance evaluation criteria

Finally, the predicted coordinates are compared to the actual coordinates. In order to enable comparison with similar studies in the literature (Okorokova et al., 2015), the squared correlation coefficient (*CC*^2^) is used as a measure of the efficiency of reconstruction. The *CC*^2^ can be calculated as follows (Nielsen et al., 2011):

(8) $$\begin{array}{l} CC^{2}=1-\frac{\sum_{i=1}^{N}{\left(\hat{s_{i}}-s_{i}\right)}^{2}}{\sum_{i=1}^{N}{\left(s_{i}-{\overset{-}{s}}_{i}\right)}^{2}} \end{array}$$

where *N* is the number of data samples within each trial, *s*_*i*_ is the *i*th x or y coordinate, $\hat{s_{i}}$ is the corresponding coordinate estimate from the GEP-based prediction model, and ${\overset{-}{s}}_{i}$ is the average of *s*_*i*_. The numerator in the second term of Equation (8) is the total mean square error of the estimates and the denominator is the total variance of the actual coordinates. The performance index defined in Equation (8) is thus a global indicator of the estimation quality of the GEP-based prediction model, as it represents the percentage of total variation of the x or y coordinates captured by the estimation.

We used formula (8) to calculate the reconstruction accuracy for X-coordinate and Y-coordinate separately. The accuracy was computed within each trial and then averaged across trials for each symbol.

### 2.3. The experiment

#### 2.3.1. Participants

This study was approved by the Ethics Committee at Donghua University and got confirmation that all experiments conform to the relevant regulatory standards. Five male volunteers (age range: 25–33 years old, height range: 169–178 cm, weight range: 62–73 kg) were recruited for this study. All participants had a medical examination to exclude upper limb musculoskeletal and nervous diseases, and they were right-handed. Before the experiment, they were requested not to participate in any intense upper-limb activities.

#### 2.3.2. Selected drawn symbols

**Figure 3:**
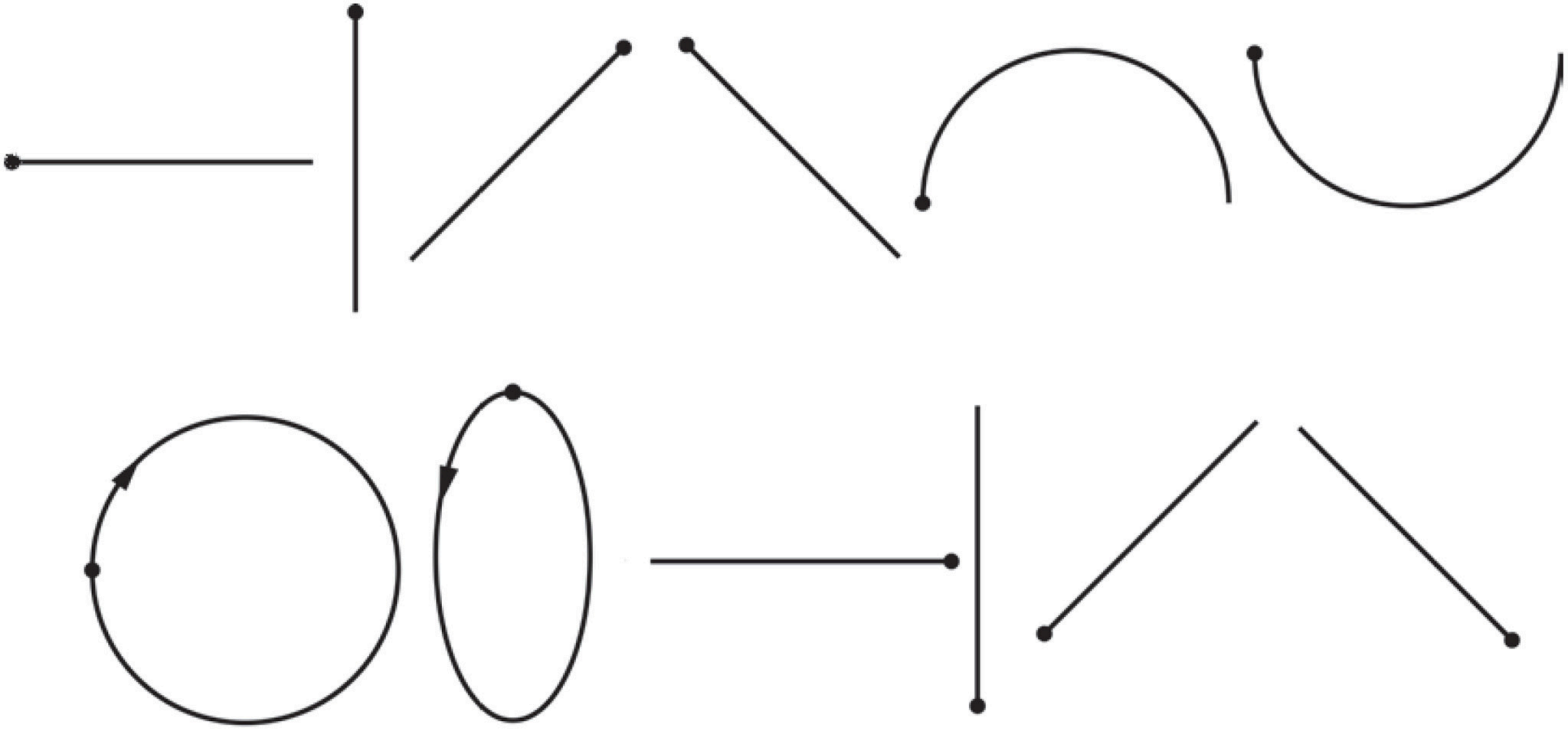
**The 12 basic one-stroke drawn shapes used in our study**. Dots represent starting points, arrows represent directions. The shapes are, from left to right, in order, horizontal line, vertical line, forward slash, backslash, arch, inverted arch, circle, ellipse, reversed horizontal line, reversed vertical line, reversed forward slash, reversed backslash.

Every complicated multi-stroke shape can be produced with an arbitrary number of single strokes, considered as a primitive of drawing and handwriting (Djioua and Plamondon, 2008). However, there is no definitive set of basic shapes (Yang and Chen, 2016). For gesture shape recognition based on computer-vision methods, the x and y position data of hand gesture traces can be converted into direction chain codes representing basic direction vectors (Yoon et al., 2001; Asano and Honda, 2010). The eight-directional chain codes (Yoon et al., 2001; Asano and Honda, 2010) can be connected continuously to represent arbitrary shapes, so eight-directional straight lines can be selected as the basic one-stroke shapes here. In addition, arch and inverted arch are also basic shapes than can be connected to form the circle, ellipse, S curves and so on, so they will be selected. Circle and ellipse can be formed by the arch and inverted arch, but they are widely-used one-stroke shapes. Thus, we will also select circle and ellipse as slightly more complicated shapes to test the feasibility of our method. In conclusion, we will select 12 basic widely-used one-stroke shapes in this exploratory experiment, although there are many single strokes of different shapes and angles during drawing. Figure 3 shows the images of twelve basic one-stroke drawn shapes used.

#### 2.3.3. Tested muscles

**Figure 4:**
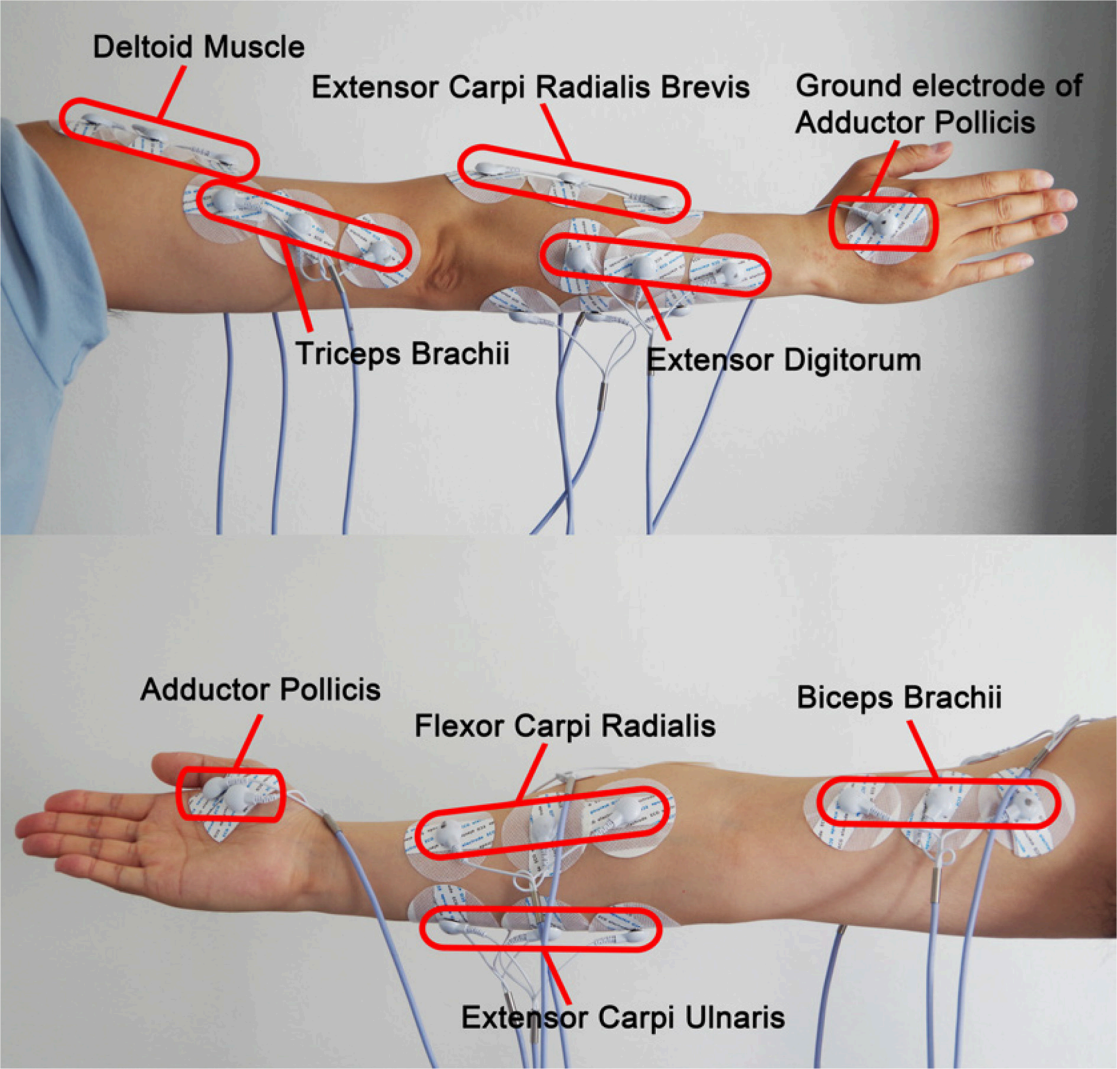
**Placement of the sEMG electrodes**. The trigger channel for determining the onset of each trial and the seven channels for feature extraction are included.

We selected 8 channels to record EMG activities from 8 muscles respectively. The locations of the electrodes are shown in Figure 4. Among them, one channel used for determining the onset of each drawing movement was set as the trigger channel, triggering the coordinate recording from the digital tablet and feature extraction of sEMG signals from the remaining seven channels simultaneously. We set the trigger channel to measure the EMG activities of the thumb over Adductor Pollicis (AP), which is the largest but deepest thenar muscle (Schmidt and Lanz, 2003) and sensitive to the movement of clicking on the starting button of the digital pen. Thus, the purpose of the trigger channel was simply to indicate the onset of the drawing trial by detecting threshold crossing, and the epoch onset of the trigger channel will be designated for all the remaining 7 channels.

We chose another 7 channels to measure EMG activities for feature extraction over four forearm muscles: Flexor Carpi Radialis (FCR), Extensor Digitorum (ED), Extensor Carpi Ulnaris (ECU), Extensor Carpi Radialis Brevis (ECRB) and three upper arm muscles: Triceps Brachii (TB), Deltoid Muscle (DM) and Biceps Brachii (BB). Since the drawing and handwriting involve the finger, wrist, and whole arm movements (Linderman et al., 2009; Robertson and Bertling, 2013), sEMG signals can be recorded from intrinsic hand, forearm and upper arm muscles that produce these movements. However, the drawing movements may arise electrode shift, which will interfere the collection of EMG signals from hand and reduce the recognition accuracy. Therefore, only EMG activity over the forearm and upper arm muscles was measured for feature extraction.

#### 2.3.4. Experimental protocol

The experiments mainly included simultaneous measurements of drawing traces and sEMG signals. Each trial for each subject began by simultaneously recording the drawing trace and the corresponding sEMG signals. Drawing traces were recorded using the digital tablet, yielding a pair of coordinates in the two-dimensional space. EMGs were recorded using the surface EMG electrodes placed on the skin overlying measured muscles.

The experiment was divided into three stages: a welcome stage, a preparation stage and a task stage. During the welcome stage, the procedures and the equipment used for the experiment were introduced to the participants. All participants were then required to sign a consent form with a detailed description of the experiment, and anthropometric measurements (age, height, weight) were made.

During the preparation stage, we printed each shape on 12 × 12 cm paper as a template and put it between the transparent photo frame and the work area of the digitizing tablet for each trial. For the task stage, we prepared 12 sheets of template paper totally, containing all the selected drawing characters.

During the task stage, after the sEMG electrodes were attached on their right arms and hands and all signals were normal, each subject comfortably seated at a desk in front of a digital tablet was requested to draw on the tablet with a pen while tracing each template. Subjects were required to click and hold down the starting button of the digital pen to trigger the recording of the x and y coordinates of the drawing trace. At the end of each trial, they were required to release the starting button to stop the coordinate recording. The EMGs of 8 muscles were simultaneously recorded during each trial.

**Figure 5:**
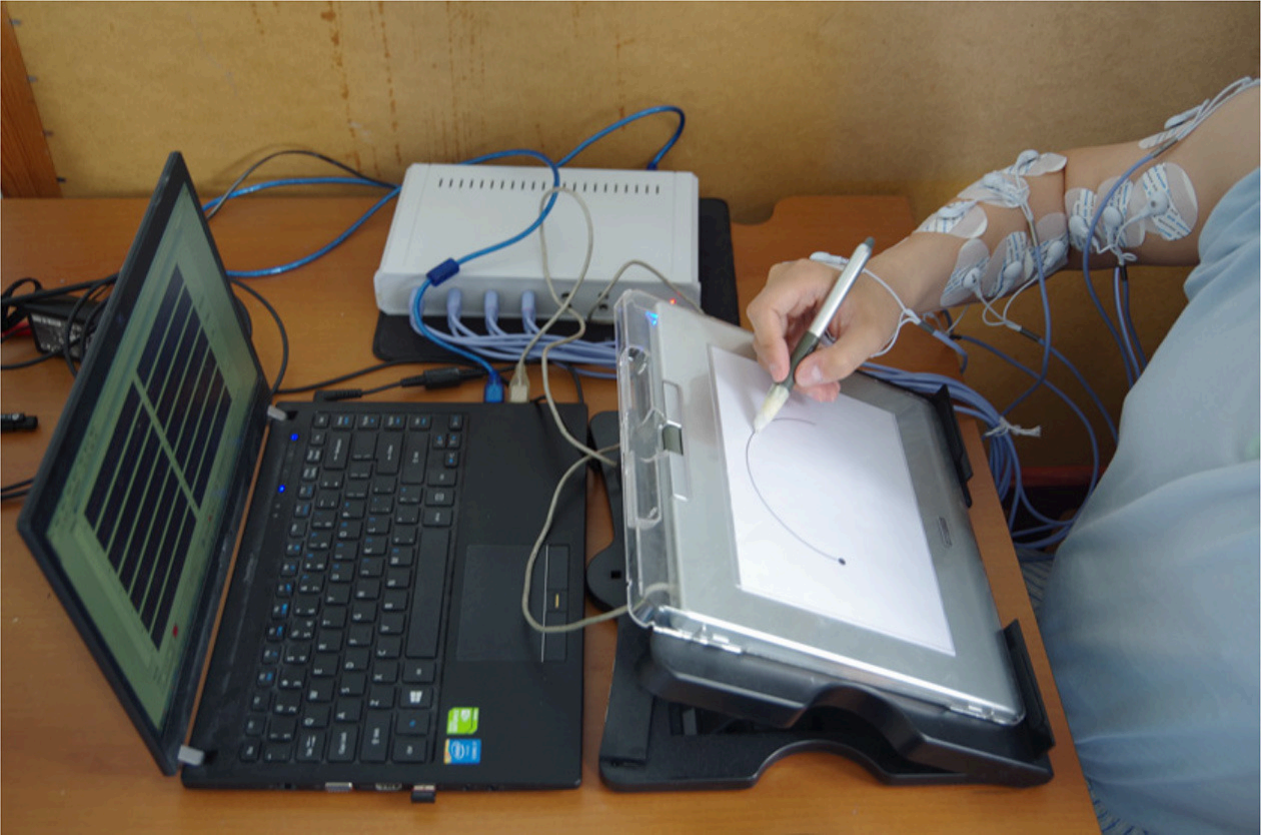
**A photograph of a recording session**.

Each participant was instructed to trace the 12 drawing templates, repeating each symbol approximately 40 times. The order of the stimuli (template) was randomized. Therefore, each subject drew 480 symbols (i.e., performed 480 trials) during a 1-day recording session. To avoid muscle fatigue, participants were asked to rest for 3 min after 10 consecutive trials. At the same time, we can change the template randomly. Participation in the experiment took each subject approximately 150 min. The procedure can be illustrated in Figure 5.

#### 2.3.5. Data acquisition

**Figure 6:**
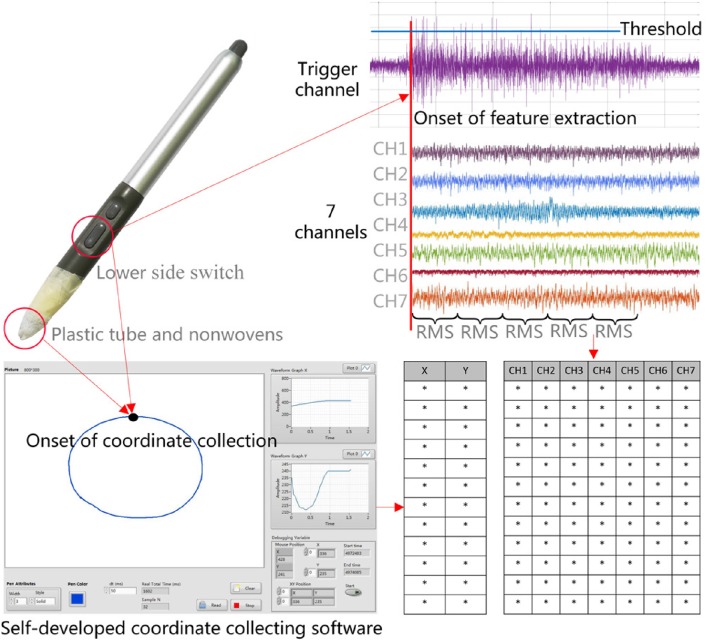
**The data acquisition scheme**.

Our system acquired data from two sources: x and y coordinates of drawing traces and sEMG signals. The data acquisition scheme is shown in Figure 6. The x and y coordinates were measured using a Graphire 4/Wacom tablet and a matched digital pen. The tablet data were sampled at 100 Hz, which included mouse click events generated when the subject pressed and held down the lower side switch (starting button) of the pen or released it. The lower side switch is set to left click. We wrapped the pen tip using nonwovens and a plastic tube with a height of 3 mm and a diameter of 3 mm to it not contact the tablet surface directly and not trigger the function of the pen tip, while still within 5 mm of the tablet surface. The reason is that when positioning the screen cursor and operating a side switch, the pen tip needs to be within 5 mm of the tablet surface. We will collect the x and y coordinates during each drawing trial every 50 ms in a self-developed coordinate collecting software (Figure 6).

A 12-channel digital EMG system (ZJE-II, ZJE Studio Ltd., China) and eight sEMG sensors (ZJE-II, ZJE Studio Ltd., China) were used to collect, amplify and transmit sEMG signals of the eight muscles in the experiment. The sensors are able to detect sEMG signals from 0 to 2,000 μV. The raw signals were sampled at 1,000 samples/s and band-pass filtered at 10–500 Hz with a notch filter implemented to remove the 50 Hz line interference.

The single disposable Ag/AgCl circular electrodes (5.7 cm in diameter), filled with conductive electrode paste (Jun Kang Medical Supplies Ltd., China), were used to measure sEMG activity. Because the test muscle of hand (Adductor Pollicis) and thenar areas do not offer enough space for these electrodes, the electrodes were cut to a smaller strip shape (5 cm in length and 3.5 cm in width) for hand muscle. The diameter of electrode itself is 1 cm. The electrode can be snapped onto the EMG cable that connects it to the EMG amplifier. Before electrode attachment, alcohol was used to clean the skin, and conductive gel was used to improve the contact of the electrode with the skin (Chen et al., 2015). Then, the pairs of triode electrodes of the sEMG sensors were attached to the eight muscles of the subjects right arm and hand. The placement of electrodes was in the direction of the muscle fibers on the midline of the muscle belly to avoid the innervation zone of the muscles (Tang et al., 2015). The raw signals were sampled at 1,000 samples/s and band-pass filtered at 10–500 Hz with a notch filter implemented to remove the 50 Hz line interference.

The 7 channels of arm muscles were measured for feature extraction and further reconstruction. The trigger channel of hand muscle was measured for determining the onset of each trial. The trigger channel will be used for detecting threshold crossing, and the epoch onset of the trigger channel will be designated for all the remaining 7 channels. The threshold of EMG amplitude was set as ≥40 μV during each test symbol. After the first value crossing the threshold, the position of the maximum peak value during the following 20 ms is determined as the epoch onset of the drawing movement.

#### 2.3.6. Two basic experimental designs

We used two basic experimental designs to calibrate our hybrid reconstruction model.

##### 2.3.6.1. Within-group design

A single set of prediction models (*F*(*x*), *F*(*y*)) was estimated using the training trials from all shapes at the same time and then tested on the remaining test trials.

##### 2.3.6.2. Between-group design

A separate set of prediction models (*F*(*x*_*n*_), *F*(*y*_*n*_), *n* ∈ 1, ⋯12) was estimated for each shape and then tested within the data from the trials of the same shape.

For Within-group design, only one set of prediction models was estimated by pooling all the training samples together, while in between-group design the x and y prediction models were estimated separately for each of the 12 shapes. Then, the out-of-training sample measurements were used to reconstruct drawing traces via the recursive process outlined in the section of coordinate state transition.

#### 2.3.7. Comparison with the Kalman filter

For comparison purposes, we used the same training and testing dataset for the Kalman Filter (KF) estimate, which was originally tested on the handwriting symbols 0–9 by Okorokova et al. (2015). The Kalman Filter approach allows to fuse two information sources: the physical characteristics of drawing and the activity of the leading hand muscles, registered by the sEMG.

In this application, the first information source is the state transition model that captures the dependence between the state vector (The differences of x and y coordinates) at time *t* and the state vectors from the past. Then the dynamical model is formalized as a multivariate autoregressive (MVAR) process, whose parameters are estimated from the data. The second source of information is the measurement model that captures the dependence between the state vector and the extracted features of seven sEMG signals. Then the relation of sEMG signals and the drawing coordinate is modeled via multivariate linear regression equation with coefficients determined from the training data-set.

##### 2.3.7.1. State transition model

As the first information source, the equation is as follows:

(9) $$\begin{array}{l} s_{i}=As_{i\;-\;1}+v_{i} \end{array}$$

where *s*_*i*_ = [Δ*x*_*i*_, Δ*y*_*i*_] is a [2 × 1] state vector containing the differences of x and y coordinates; *A* is a [2 × 2] state transition matrix; *v*_*i*_ is a [2 × 1] vector containing process noise, which is assumed to be drawn from a multivariate Gaussian distribution with zero mean and covariance matrix *Q*.

##### 2.3.7.2. Measurement model

The equation of the second information souce is as follows:

(10) $$\begin{array}{l} s_{i}=Hz_{i}+w_{i} \end{array}$$

where *z*_*i*_ is a [7 × 1] observation vector containing 7 extracted RMS measurements; *H* is a [2 × 7] measurement transformation matrix; *w*_*i*_ is a [7 × 1] vector of measurement noise with zero mean and covariance matrix *R*.

##### 2.3.7.3. The kalman filter algorithm

Here we can formulate the algorithm for calculating the Kalman Filter estimate. The computation can be split into three consecutive steps.

Step 1. Endogenous state prediction and error covariance update:
  (11) $$\begin{array}{l} {ŝ}_{i|i\;-\;1}=\mu_{1i}=A{ŝ}_{i\;-\;1|i\;-\;1} \end{array}$$
  (12) $$\begin{array}{l} P_{i|i\;-\;1}=\Sigma_{1i}=AP_{i\;-\;1|i\;-\;1}A^{T}+Q \end{array}$$
Step 2. Kalman Gain Calculation:
  (13) $$\begin{array}{l} K_{i}=\Sigma_{1i}{\left(\Sigma_{1i}+\Sigma_{2i}\right)}^{-1}=P_{i|i\;-\;1}{\left(P_{i|i\;-\;1}+R\right)}^{-1} \end{array}$$
Step 3. Measurement Update:
  (14) $$\begin{array}{l} {ŝ}_{i|z}=\mu_{2i}=Hz_{i} \end{array}$$
  (15) $$\begin{array}{l} {ŝ}_{i|i}={ŝ}_{i|i\;-\;1}+K_{i}\left({ŝ}_{i|z}-{ŝ}_{i|i\;-\;1}\right) \end{array}$$
  (16) $$\begin{array}{l} P_{i|i}=P_{i|i\;-\;1}-K_{i}P_{i|i\;-\;1} \end{array}$$

##### 2.3.7.4. Training and testing

The training trials are used for learning of the parameters of the state transition model (matrices A and Q) and the measurement model (matrices H and R). We applied Ordinary Least Squares Method to estimate matrix A in the state transition equation and matrix H in the measurement equation. Covariance matrices R and Q were estimated based on the residuals of the two fitted models. The RMS data from the testing trials and the learned matrices are then used for the prediction of the state vector in both models. Finally, the predictions of the two models are merged via the Kalman filter algorithm and the result of the filter is compared to the actual state vector. The squared correlation coefficient (*CC*^2^) is used as a measure of efficiency of reconstruction. More detailed description of Kalman Filter can be found in Okorokova et al. (2015). The Kalman Filters in this paper were performed using Matlab R2016a (The MathWorks Inc., Natick, USA).

## 3. Results

Reconstruction models were trained and tested on the data across subjects. After feature extraction, the dataset was randomly divided into two subsets, a training set and test set, for reconstruction. Seventy percent of the data were selected as the training set and 30% as the test set.

### 3.1. Results of within-group design

In the within-group design, we first trained one set of difference prediction models (*F(x), F(y)*) for all drawn shapes and used it to reconstruct drawing traces from the sEMG data in the testing set. In this run, the best fitness by the GEP model of X-coordinate was found with *f*_*i*_ = 94.848 and RMSE = 9.543. The best fitness by the GEP model of Y-coordinate was found with *f*_*i*_ = 87.546 and RMSE = 10.423. The deduced formula for X-coordinate is as given below:

(17) $$\begin{array}{l} F\left(x\right)=\sin\left(d_{5}-d_{1}-\frac{d_{5}}{d_{4}}+d_{4}-1.74+\frac{2.09d_{2}-4.37}{d_{5}}\right)\\ +\;4.3+d_{7}-d_{1}+\frac{d_{7}}{\exp\left({\left(\exp\left(\frac{\cos\left(d_{1}\;+\;d_{4}\;+\;42.63\right)}{d_{7}}\right)\right)}^{2}\right)}\\ -\;\frac{d_{2}\sin\left(d_{3}+8.89-d_{5}\right)}{d_{7}}-d_{4}-\\ \frac{\exp\left(\sin\left(\sqrt{d_{1}}\right)\right){\left(d_{2}-3.53\right)}^{2}}{62.73}\\ +\;\cos\left(2d_{1}-0.45+\frac{1}{d_{3}}\right)+\sin\left(d_{7}\right)\\ +\;\cos\left(d_{1}+7.63-\cos\left(\cos\left(d_{3}\right)+2d_{5}\right)-d_{5}\right)\\ -\;d_{6}+d_{2}+d_{5}-d_{3} \end{array}$$

where *d*_1_ is *RMS* of ECRB, *d*_2_ is *RMS* of ED, *d*_3_ is *RMS* of ECU, *d*_4_ is *RMS* of FCR, *d*_5_ is *RMS* of TB, *d*_6_ is *RMS* of BB, *d*_7_ is *RMS* of DM.

The deduced formula for Y-coordinate is as given below:

(18) $$\begin{array}{l} F\left(y\right)=\frac{d_{1}\sin\left(d_{1}\right)}{\exp\left(\sin\left(-0.36d_{6}\right)+12.21d_{2}\right)}\\ -\;2d_{6}+2d_{1}+d_{7}-sin\left(d_{2}+0.96\right)\\ +\;\sin\left(d_{1}-d_{5}-{\left(\exp\left(\frac{-0.78d_{5}}{d_{3}}+\sin\left(d_{7}\right)\right)\right)}^{2}\right)\\ +\;cos\left({\left(\frac{d_{3}-d_{6}+6.67}{-0.09d_{2}}\right)}^{2}\right)-8.33+\frac{1}{d_{7}} \end{array}$$

where the input dimension of RMS of FCR (*d*_4_) was removed in the formula after training.

**Figure 7:**
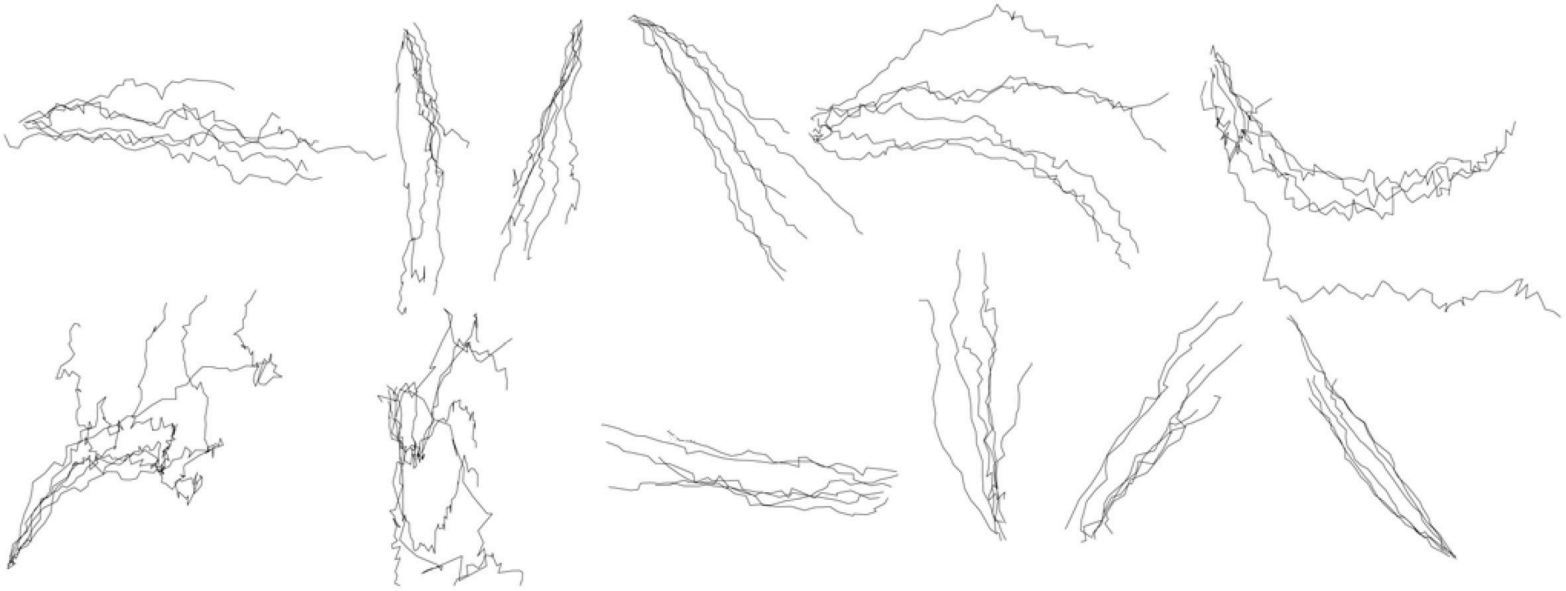
**Within-group reconstruction using the three-step hybrid model: several randomly selected reconstructed trials of each shape across five participants**. The shapes are, from left to right, in order, horizontal line, vertical line, forward slash, backslash, arch, inverted arch, circle, ellipse, reversed horizontal line, reversed vertical line, reversed forward slash, reversed backslash.

A detailed accuracy distribution for all shapes is shown in Table 2. Firstly, the accuracy of reconstruction for each test trial available for each shape was calculated, then the statistics within trials of the same shape to determine the mean and standard deviation across 5 participants were obtained. Reconstruction accuracy by coordinates independently, and an average between the two coordinates are reported. Figure 7 shows the result of the reconstruction of several trials of each shape for one of the participants. The drawing shapes are identifiable, despite the circle being noisy and inaccurate. As can be seen, we managed to achieve the average accuracy of 79 ± 25 and 70 ± 31% for the two reconstructed coordinates, as estimated for the 5 participants of the experiment.

**Table 2:** **Within-group reconstruction performance using the three-step hybrid model: the average reconstruction accuracy of each shape across the 5 participants**.

**Average performance, *CC*^2^(mean ± standard deviation)**			
**Shape**	**X-coordinate**	**Y-coordinate**	**Average: (X + Y)/2**
Horizontal line	0.99 ± 0.007	0.62 ± 0.31	0.81 ± 0.26
Vertical line	0.84 ± 0.13	0.98 ± 0.03	0.91 ± 0.09
Forward slash	0.69 ± 0.32	0.97 ± 0.07	0.82 ± 0.2
Backslash	0.98 ± 0.017	0.99 ± 0.01	0.98 ± 0.006
Arch	0.98 ± 0.013	0.4 ± 0.23	0.69 ± 0.13
Inverted arch	0.96 ± 0.04	0.34 ± 0.23	0.64 ± 0.18
Circle	0.19 ± 0.13	0.2 ± 0.11	0.19 ± 0.01
Ellipse	0.56 ± 0.15	0.41 ± 0.28	0.48 ± 0.1
Reversed horizontal line	0.97 ± 0.039	0.48 ± 0.31	0.72 ± 0.16
Reversed vertical line	0.54 ± 0.29	0.98 ± 0.03	0.76 ± 0.15
Reversed forward slash	0.8 ± 0.23	0.98 ± 0.019	0.89 ± 0.13
Reversed backslash	0.96 ± 0.03	0.99 ± 0.013	0.98 ± 0.02
All	0.79 ± 0.25	0.7 ± 0.31	0.74 ± 0.22

### 3.2. Results of between-group design

**Figure 8:**
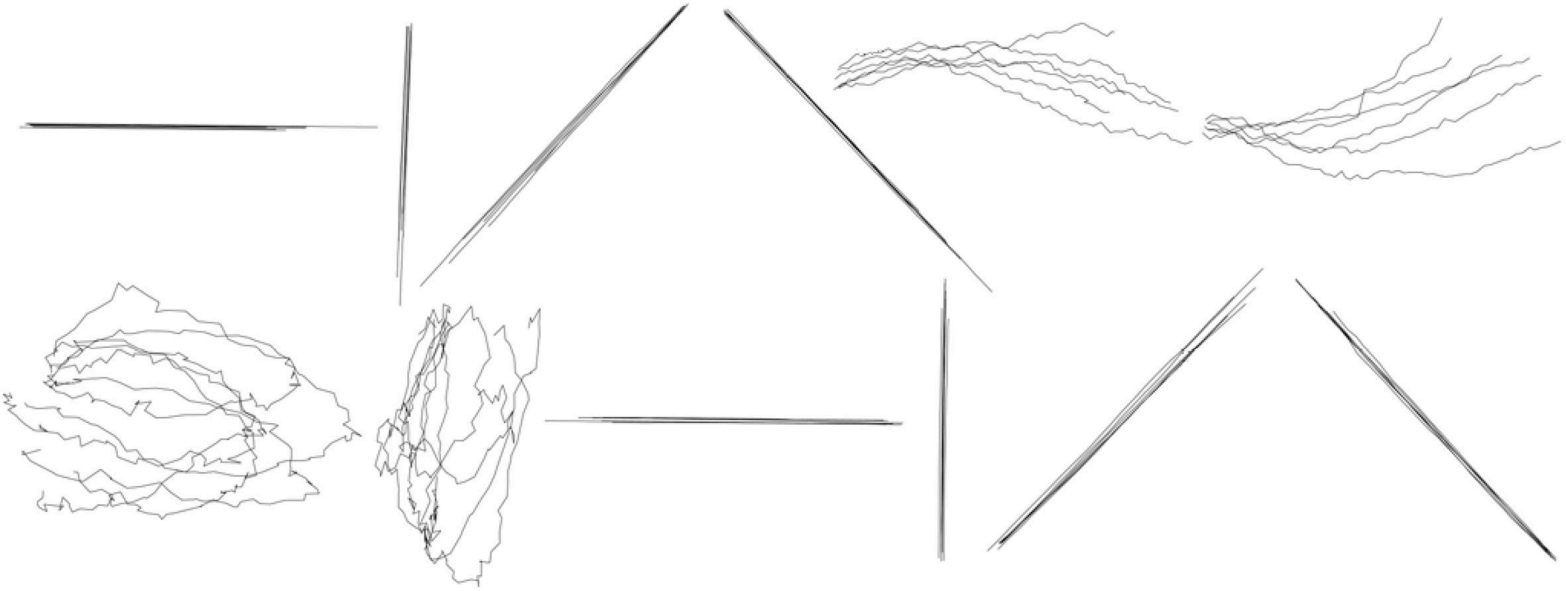
**Between-group reconstruction using the three-step hybrid model: several randomly selected reconstructed trials of each shape across five participants**. The shapes are, from left to right, in order, horizontal line, vertical line, forward slash, backslash, arch, inverted arch, circle, ellipse, reversed horizontal line, reversed vertical line, reversed forward slash, reversed backslash.

In the between-group design, the x-coordinate and y-coordinate prediction models were constructed for each shape separately, and then the traces of the same shape were reconstructed. We can construct in total 12 prediction models for the x-coordinate of each shape and 12 prediction models for the y-coordinate of each shape. Figure 8 shows the results of reconstruction of several trials of each symbol across participants. The separate GEP-based prediction models for each shape perform a more specific and accurate reconstruction than the unified GEP-based prediction models, which is visually evident from the comparison of Figures 7, 8.

Table 3 shows the average reconstruction accuracy of each shape with separate GEP-based prediction models. The average reconstruction accuracy in the two coordinates across subjects was 91 ± 13 and 81 ± 7 respectively, which is higher than that of within-group reconstruction.

**Table 3:** **Between-group reconstruction performance using the three-step hybrid model: the average reconstruction accuracy of each shape across the 5 participants**.

**Average performance, *CC*^2^(mean ± standard deviation)**			
**Shape**	**X-coordinate**	**Y-coordinate**	**Average: (X + Y)/2**
horizontal line	0.99 ± 0.002	0.74 ± 0.28	0.87 ± 0.18
vertical line	0.91 ± 0.06	0.99 ± 0.002	0.95 ± 0.06
Forward slash	0.99 ± 0.006	0.93 ± 0.21	0.96 ± 0.04
Backslash	0.99 ± 0.004	0.99 ± 0.004	0.99 ± 0.004
Arch	0.97 ± 0.06	0.67 ± 0.28	0.82 ± 0.21
Inverted arch	0.98 ± 0.007	0.53 ± 0.33	0.75 ± 0.32
Circle	0.78 ± 0.24	0.65 ± 0.3	0.72 ± 0.09
Ellipse	0.66 ± 0.27	0.67 ± 0.29	0.66 ± 0.002
Reversed horizontal line	0.99 ± 0.06	0.5 ± 0.32	0.74 ± 0.16
Reversed vertical line	0.6 ± 0.33	0.99 ± 0.006	0.78 ± 0.29
Reversed forward slash	0.99 ± 0.003	0.99 ± 0.003	0.99 ± 0.0004
Reversed backslash	0.99 ± 0.003	0.99 ± 0.003	0.99 ± 0.0005
All	0.91 ± 0.14	0.81 ± 0.18	0.86 ± 0.12

### 3.3. Results of the Kalman filter

#### 3.3.1. Results of the within-group design

**Figure 9:**
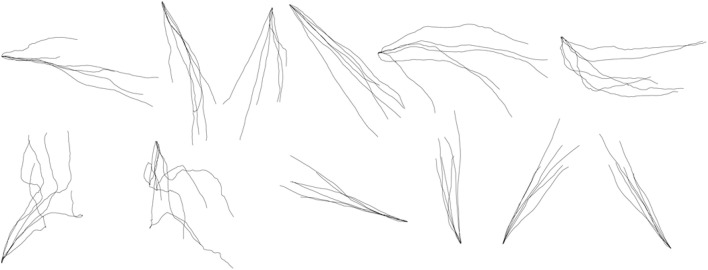
**Within-group reconstruction using the Kalman Filter: several randomly selected reconstructed trials of each shape across five participants**. The shapes are, from left to right, in order, horizontal line, vertical line, forward slash, backslash, arch, inverted arch, circle, ellipse, reversed horizontal line, reversed vertical line, reversed forward slash, reversed backslash.

We reconstructed drawing traces by Within-group design (one set of parameters for all symbols), using the Kalman Filter. A single set of parameters (*A, H, R, Q*) was estimated using the training trials from all shapes at the same time and then tested on the remaining test trials. A detailed accuracy distribution for all shapes is shown in Table 4. Figure 9 shows the result of the reconstruction of six trials of each shape for one of the participants.

**Table 4:** **Within-group reconstruction performance using the Kalman Filter: the average reconstruction accuracy of each shape across the 5 participants**.

**Average performance, *CC*^2^(mean ± standard deviation)**			
**Shape**	**X-coordinate**	**Y-coordinate**	**Average: (X + Y)/2**
Horizontal line	0.98 ± 0.01	0.71 ± 0.26	0.85 ± 0.13
Vertical line	0.35 ± 0.27	0.98 ± 0.02	0.67 ± 0.14
Forward slash	0.93 ± 0.11	0.98 ± 0.01	0.96 ± 0.05
Backslash	0.98 ± 0.01	0.98 ± 0.01	0.98 ± 0.008
Arch	0.94 ± 0.033	0.41 ± 0.15	0.68 ± 0.07
Inverted arch	0.86 ± 0.21	0.24 ± 0.21	0.55 ± 0.15
Circle	0.32 ± 0.26	0.42 ± 0.08	0.37 ± 0.14
Ellipse	0.40 ± 0.23	0.14 ± 0.15	0.27 ± 0.13
Reversed horizontal line	0.92 ± 0.19	0.43 ± 0.31	0.67 ± 0.18
Reversed vertical line	0.59 ± 0.32	0.98 ± 0.02	0.78 ± 0.16
Reversed forward slash	0.86 ± 0.24	0.98 ± 0.01	0.92 ± 0.12
Reversed backslash	0.96 ± 0.03	0.99 ± 0.004	0.98 ± 0.01
All	0.76 ± 0.16	0.7 ± 0.1	0.72 ± 0.11

According to Tables 2, 4, the within-group reconstruction performance attained with our method (79 ± 25 and 70 ± 31% for the two coordinates, respectively) is comparable to that achieved by the Kalman Filter (76 ± 16 and 70 ± 10% for the two coordinates, respectively).

#### 3.3.2. Results of the between-group design

**Figure 10:**
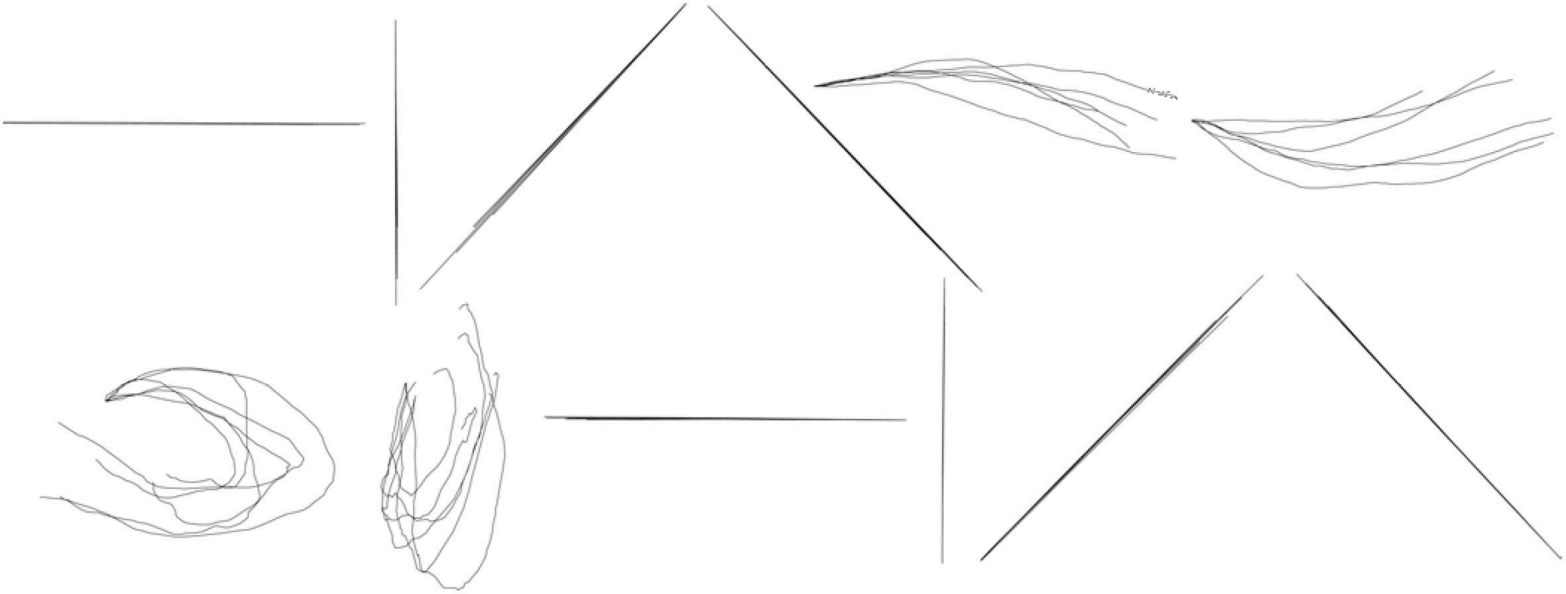
**Between-group reconstruction using the Kalman Filter: several randomly selected reconstructed trials of each shape across five participants**. The shapes are, from left to right, in order, horizontal line, vertical line, forward slash, backslash, arch, inverted arch, circle, ellipse, reversed horizontal line, reversed vertical line, reversed forward slash, reversed backslash.

During the between-group design, a separate set of parameters (*A*_*n*_, *H*_*n*_, *R*_*n*_, *Q*_*n*_, *n* ∈ 1, ⋯12) was estimated for each symbol and then tested within the data from the trials of the same symbol. Figure 10 shows the results of reconstruction of several trials of each symbol across participants. Table 5 shows the average reconstruction accuracy of each shape with separate GEP-based prediction models. According to Tables 3, 5, the between-group reconstruction performance attained with our method (91 ± 14 and 81 ± 18 for the two coordinates, respectively) is also comparable to that achieved by the Kalman Filter (85 ± 9 and 82 ± 14% for the two coordinates, respectively).

**Table 5:** **Between-group reconstruction performance using the Kalman Filter: the average reconstruction accuracy of each shape across the 5 participants**.

**Average performance, *CC*^2^(mean ± standard deviation)**			
**Shape**	**X-coordinate**	**Y-coordinate**	**Average: (X + Y)/2**
horizontal line	0.99 ± 0.003	0.76 ± 0.25	0.88 ± 0.12
vertical line	0.45 ± 0.25	0.99 ± 0.004	0.72 ± 0.12
Forward slash	0.98 ± 0.006	0.99 ± 0.005	0.99 ± 0.006
Backslash	0.99 ± 0.004	0.99 ± 0.004	0.99 ± 0.004
Arch	0.97 ± 0.01	0.64 ± 0.3	0.81 ± 0.15
Inverted arch	0.97 ± 0.006	0.59 ± 0.31	0.78 ± 0.15
Circle	0.74 ± 0.26	0.74 ± 0.26	0.74 ± 0.16
Ellipse	0.60 ± 0.28	0.71 ± 0.29	0.65 ± 0.18
Reversed horizontal line	0.98 ± 0.007	0.47 ± 0.33	0.72 ± 0.16
Reversed vertical line	0.59 ± 0.32	0.98 ± 0.007	0.79 ± 0.16
Reversed forward slash	0.98 ± 0.004	0.98 ± 0.004	0.98 ± 0.004
Reversed backslash	0.99 ± 0.003	0.99 ± 0.003	0.99 ± 0.003
All	0.85 ± 0.09	0.82 ± 0.14	0.84 ± 0.11

As we can see from the Figures 7–10, the Kalman Filter has the ability to smooth the noise and, as a result, provide a more smoother, comprehensible and realistic reconstruction than our method.

## 4. Discussion

In this work, we applied the novel three-step hybrid model to the reconstruction of drawing traces on the basis of sEMG measurement. To our knowledge, this study is the first to use the GEP to predict x and y coordinates from sEMG signals of arm muscles. The results of this study demonstrate that our three-step hybrid model performs very well.

It can be observed that the hybrid model combines three-step algorithms to reconstruct drawing traces. The first step is coordinate state transition, the second step is feature extraction of sEMG signals, and the third step is to construct prediction algorithms derived by GEP.

The first two steps reveal the basis of the excellent prediction results of prediction models derived by GEP. In the first step, the differences between the *i*th coordinate state and the *i*−1th coordinate state set as the target vectors of GEP, instead of original x and y coordinates, has following three advantages: (1) Compared with original x and y coordinates, the difference of coordinates is more associated with the dynamical emerging procedure of sEMG signals (Stegeman et al., 2000). The surface EMG is obtained by convolution of each motor neuron spike train by the motor unit action potential (Farina et al., 2014). From the perspective of physiology, sEMG is the electrical manifestation of the contracting muscles activity from the drawing movements (Naik et al., 2007). From the perspective of coordinates, the differences between the present coordinate state and the previous state can also reflect the dynamic drawing movements. (2) The difference of coordinates can reduce the dimensionality of target vectors and provide a limited range of values within a norm. Thus, reconstruction accuracy can be improved by making fewer classes (target vectors) available to the GEP algorithm (Earley et al., 2016). (3) The differences of coordinates set as target vectors can make our reconstruction procedures operate causally. The difference is calculated from the present coordinate and the immediately past coordinate. In the problem of reconstruction of handwriting from multichannel EMG activity, the Kalman filter (Okorokova et al., 2015) that operates in a causal manner gets higher average accuracy in both coordinates than that found by Linderman et al. (2009), where non-causal Wiener Filter based reconstruction was employed. The concept of Kalman filter happened to coincide with our method. In the second step, to design a well performed GEP-based sEMG reconstruction system, the feature plays a critical role (Xing et al., 2014). The adjacent analysis windows have a duration of 50 ms for feature (RMS) extraction, which make the RMS values (input vectors) more associated with the differences (target vectors) calculated from the x and y coordinates collected every 50 ms.

In the third step, using the GEP, we can deduce the non-linear x and y difference prediction models with high accuracy and generalizability from target and input vectors collected in the first and second steps. In the within-group design, the GEP model further reduced the seven input dimensions to six (*d*_1_, *d*_2_, *d*_3_, *d*_5_, *d*_6_, *d*_7_) in y-coordinate prediction model after training. We can conclude that the removed input dimension (*d*_4_), representing RMS values of Flexor Carpi Radialis, is irrelevant to the differences of y-coordinate. However, there were no input dimensions reduced in x-coordinate prediction model after training. Thus, these tested muscles are leading muscles for investing the EMG-drawing relationship in further study.

The accuracy of our method is comparable to the accuracy of the Kalman Filter (KF), which allows fusing not only the EMG activity but also the physical properties of drawing. Interestingly, the reconstruction performance of our study is higher than KF in x-coordinate prediction but slightly lower in y-coordinate prediction. Also, the GEP based hybrid reconstruction model yields a mean accuracy of 74% in within-group design and 86% in between-group design, averaged for the reconstructed x and y coordinates, which is slightly outperforms that of the Kalman filter (a mean accuracy of 72% in within-group design and 84% in between-group design). Our method and the KF significantly outperform previously proposed method based on the Wiener filter (Linderman et al., 2009). The KF and Wiener filter are both belonging to linear regression methods. Our non-linear models derived by advanced evolutionary computation method (i.e., GEP) is expected to outperform the linear regression methods, because of the non-linear nature of the relation between the recorded EMG signals and the actuator trajectory.

In the novel three-step hybrid model, we purely constructed the non-linear regression models of EMG signals and drawing coordinates using GEP, without combining the physical characteristics of drawing like KF, but the results are encouraging. In future work, if we can learn from KF to take into account the dynamic model of the pen coordinate process to smooth the noise, it is estimated that the accuracy of reconstruction of the hybrid model may increase, and even go above 90%.

Drawing is freeform, but multi-stroke drawings can be made up of an arbitrary number of these basic one-stroke shapes. Thus, although the selected drawn symbols look like simpler than the numeric characters studied by Okorokova et al. (2015), this research has great application potential in drawing reconstruction from EMG.

Interestingly, the reconstruction performance of the hybrid model with the same shapes, but different starting point and direction of movement (e.g., vertical line and reversed vertical line, Tables 2, 3) is different. This is quite unexpected. However, drawing the same shape with different starting point and direction of movement can induce different hand gestures. There is no doubt that different hand gestures can yield different muscle movements and EMG signals. This leads to the different performance of the method with the same shapes, but different starting point and direction of movement.

We propose a new method to indicate the onset of the drawing trial by the trigger channel that directly detected the threshold crossing of the EMG activities of the Adductor Pollicis when the subject pressed the lower side switch of the digital pen, instead of pressure sensitive piezo film that indicated the onset of the writing session by generating a triggering pulse sent to one channel of the EMG amplifier (Linderman et al., 2009) when pen contact was detected with the film attached to the tablet. This method can simplify our system to acquire data from only two sources rather than three sources in previous studies (Linderman et al., 2009; Okorokova et al., 2015). Moreover, their method of onset detecting highly depends on the pressure sensitive piezo film. However, our method can detect the onset of the drawing movement through pressing the starting button of one pen. Thus, our method is more natural for practical applications.

The good performance of the method for both within-group design and between-group design models reveals its potential for a wide range of applications. The within-group design model can be used directly without prior information about the class of the shape, and is more applicable to on-line drawing reconstruction. The between-group design model can be applied as a second-stage algorithm after another algorithm (such as Hidden Markov Model) is used to classify the shapes into groups.

The starting pen location point $\left({\hat{x}}_{0},{\hat{y}}_{0}\right)$ is set to the x and y coordinates of the start point of each stroke, instead of zero vector. By this way, one-stroke shapes can be produced in desired positions, and multi-stroke symbols can be precisely drawn with an arbitrary number of these basic one-stroke shapes, which is important for practical applications.

The inter-subject variety can be reduced by specifying the drawing process of shapes, the starting point, and the direction of movements and requiring subjects to trace the 12 drawing templates with fixed dimensions. This makes the problem rather simplified as compared to a real-life scenario, in which people have their own way of drawing the same closed shape. Thus, the proposed hybrid model can be performed on the data across subjects. For example, in within-group design, only one set of x and y models was derived by the GEP. The training and testing of algorithms for all the participants are much more natural, feasible, and time-saving for the real-life on-line scenario than that for each individual subject independently.

Nevertheless, our findings and the general approach have several limitations:

1. It can be observed that the reconstruction performance of closed shapes (circle and ellipse) is inferior. Thus, we can deduce the x and y reconstruction models from the basic one-stroke shapes that are not closed but can form closed shapes with an arbitrary number of single strokes.
2. We have tested the common version and reversed version of the horizontal line, vertical line, forward slash, and backslash. Although the final shapes produced from the two version are the same, the starting point and the direction of movements are different between the two versions. These two versions are both frequently used in our daily life. However, the number of tested shapes in this paper is not enough for clinical applications. Therefore, in the future work, we will get and test a more completed set of basic drawing shapes with different starting points, and directions of movements to cater for all drawing habits.
3. In our previous research, we have studied sEMG-based handgrip force predictions (Yang et al., 2016). Force also needs to be considered in drawing, because it has a significant influence on the shades of strokes. In our future work, we will investigate EMG-force relationship during drawing. The force recognition from EMG signals can be the first step of the whole intelligent system, and it can be used to decide the shades of strokes.
4. We used only GEP for drawing trace reconstruction from EMG signals. In future studies, we plan to use other state-of-the-art algorithms to see whether the performance of our hybrid model can be further improved.
5. We only used the RMS values of the sEMG as input vectors to the GEP. To further improve the recognition rate, some other time and frequency domain indices of the sEMG, such as average EMG amplitude, mean absolute value, and wavelet, could be additionally used.
6. We only used the adjacent windowing techniques for feature extraction. In future work, we will try to use overlapping windowing techniques to improve the reconstruction performance.
7. Only five subjects were tested in our experiment. In future research, we plan to test the proposed hybrid model on more subjects.

## 5. Conclusion

In this paper, we have demonstrated that the three-step hybrid model based on GEP can reconstruct the drawing traces from the sEMG signal with an encouraging performance. We showed that the GEP based hybrid reconstruction model slightly outperforms the Kalman filter and. Our method is suitable for real-time applications as rather simple mathematical formulas were found by GEP with great accuracy. The first two steps of the hybrid model reveal the basis of the excellent prediction results of prediction models derived by GEP. In future work, we will learn from Kalman filter to take into account not only the EMG activity but also the physical properties of drawing to increase the reconstruction performance of the hybrid model based on GEP. Further progress in this field would potentially be introduced to many rapidly expanding practices and fields, including drawing in the air, computer-aided design, virtual reality, rehabilitation engineering, robot control, Internet of Things, as well as human-machine interfaces in general.

## Author contributions

YC helped in algorithm development; collecting, analyzing, and interpreting the data; drafting the manuscript. ZY helped in conceiving the study concept; algorithm development; collecting, analyzing, and interpreting the data; drafting the manuscript and obtaining funding.

### Conflict of interest statement

The authors declare that the research was conducted in the absence of any commercial or financial relationships that could be construed as a potential conflict of interest.
